# The protein partitioning model of body composition

**DOI:** 10.1007/s12064-026-00497-4

**Published:** 2026-11-06

**Authors:** Douglas Roy, Jody Roy

**Affiliations:** 1 University of New South Wales, Level 4, Matthews Building, Sydney, Australia; 2 Western Sydney University, Penrith, Australia

**Keywords:** Protein, Muscle, Energy Partitioning, Insulin Sensitivity, Body Composition, Rational Addiction, Resistance Training, Obesity

## Abstract

Two leading paradigms dominate discussions of obesity: the Energy Balance Model (EBM), which attributes weight gain to a surplus of caloric intake over expenditure, and the Carbohydrate-Insulin Model (CIM), which emphasizes carbohydrate-driven hormonal shifts that promote fat storage. Here we introduce the Protein Partitioning Model (PPM), a muscle-centric framework proposing that dietary protein and muscular activity interact to influence energy partitioning and thereby shape body composition even under constant caloric intake. By integrating PPM with the Rational Addiction framework from economics, we model how food choices and body composition co-evolve over time through their effects on lean and fat tissue. The model predicts that, when paired with resistance exercise, protein intake supports functional lean mass, which in turn enhances insulin sensitivity and increases the marginal utility of protein consumption. This creates a virtuous feedback loop characterized by bounded escalation, converging on stable states with relatively low adiposity. In contrast, carbohydrate-consumption feedback can produce relatively volatile, binge-prone trajectories with much higher steady-state adiposity. By treating muscle as an active regulator of nutrient partitioning rather than a passive consequence of energy balance, PPM unifies key features of both existing paradigms while suggesting a proactive, muscle-centric approach to obesity prevention and long-term metabolic health.

## Aims and premise

Obesity is a worldwide health challenge whose underlying causes and cures remain widely debated. Two broad classes of models have dominated both technical and popular discussions of obesity. The first is the Energy Balance Model (EBM), which explains weight gain as primarily due to excess caloric intake relative to expenditure (Swinburn et al. 2011). The second comprises a family of fuel-partitioning models, of which the Carbohydrate-Insulin Model (CIM) is the best known. CIM argues that consumption of carbohydrates elevates insulin secretion, leading to increased fat storage and distortions in the way the body partitions energy (Ludwig and Ebbeling 2018). Public health guidelines largely follow the EBM, recommending eating less and exercising more as primary strategies for combating obesity, while the CIM appears to be rising in popularity in public discussions and the media surrounding body composition and health issues more broadly.

This paper describes the Protein Partitioning Model (PPM) of body composition. PPM shares with fuel-partitioning models the premise that the way energy is partitioned among tissues influences subsequent metabolic state and feeding behaviour. In a nutshell, we propose that both energy balance and nutrient partitioning are crucially regulated by interactions between muscle and protein intake. In this way, PPM incorporates causal features of both energy-balance and fuel-partitioning accounts within a common theoretical framework, but unlike CIM and EBM, PPM emphasizes the role of muscle maintenance and dietary protein consumption as critical factors in obesity development and metabolic health. Whether PPM ultimately provides greater explanatory and predictive power than existing models is an empirical question. Here we show that it generates a coherent set of qualitative predictions while offering a unified account of feeding behaviour, body composition dynamics, and metabolic health that incorporates important features of both existing paradigms.

## Background

### Models of obesity and fuel partitioning

**Fig. 1:**
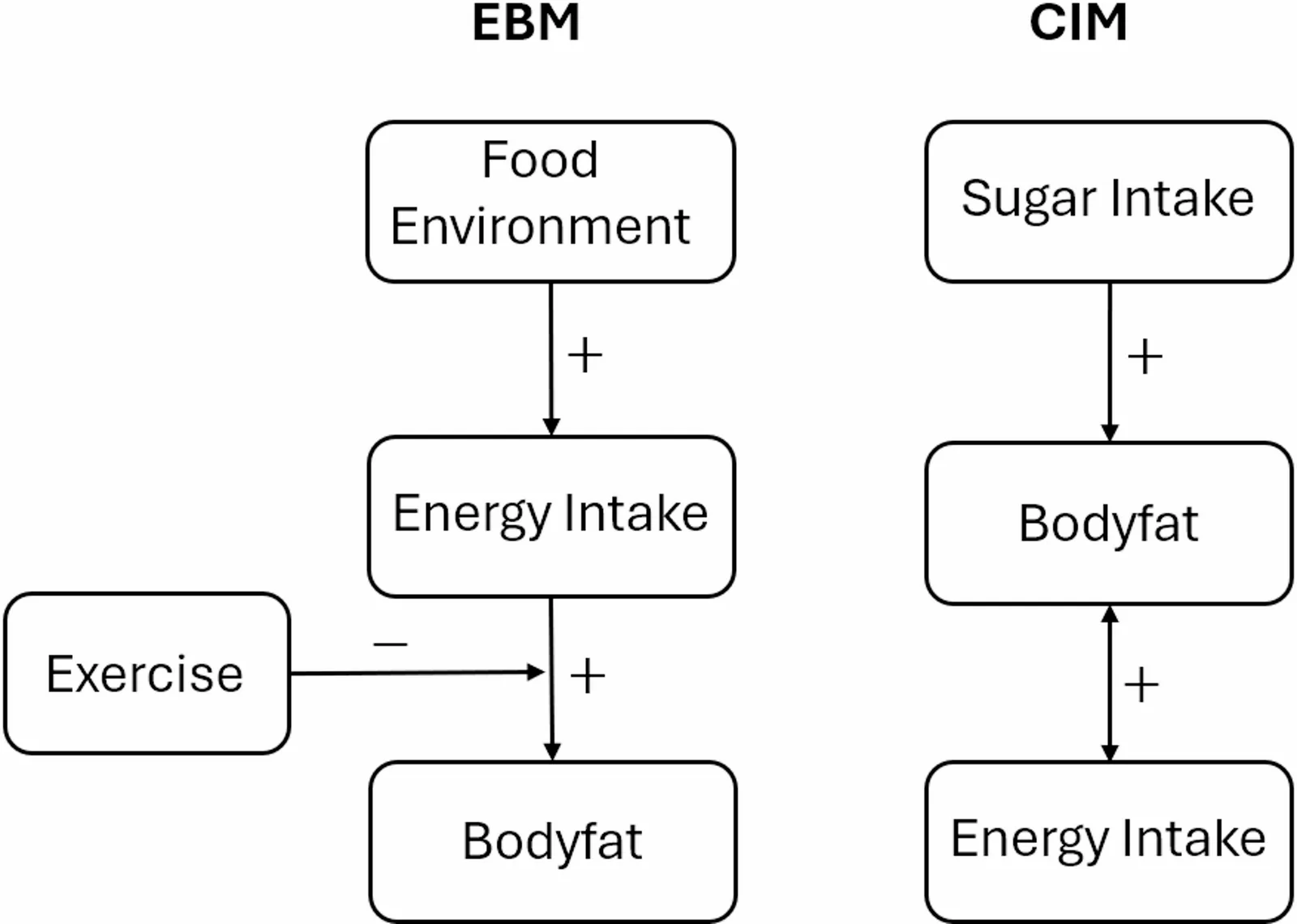
EBM and CIM compared. EBM accounts for obesity as due to changes in the food environment (e.g., abundant high energy foods and associated cues) stimulating feeding responses that lead to more energy being consumed, with the gradual accrual of energy surpluses culminating in more body fat. Exercise can reduce these surpluses by increasing energy expenditure. CIM accounts for body fat as arising from changes in nutrient partitioning caused by dietary composition (particularly sugar intake), with increased adiposity subsequently altering metabolic signals that influence appetite and energy intake. This creates a feedback loop in which changes in fuel partitioning and energy intake can reinforce one another. Note that this comparison is conceptually related to the push–pull distinction used by Ludwig and Sørensen (2022), although the present figure is intended as a pedagogical comparison of EBM and CIM considered separately rather than an integrated push–pull model. Positive (+) and inhibitory (−) influences are indicated where relevant to clarify the causal structure of each model

EBM is the most widely accepted and intuitive framework for understanding shifts in body composition. This explains changes in body fat as arising from the difference between energy consumed through food and energy expended through physiological processes and activity (Swinburn et al. 2011). According to this perspective, the macronutrient source of calories—whether from fats, protein, carbohydrates, or alcohol — is not important for body weight regulation when total caloric intake is held constant (Hall et al. 2016). CIM argues that carbohydrate intake plays a more significant role than calorie content alone by triggering insulin secretion that promotes fat storage (Ludwig and Ebbeling 2018). Figure 1 provides a simplified comparison of EBM and CIM (for an extensive review of their differences see (Magkos et al. 2024). The debate between proponents of EBM and CIM has been vigorous both in academic literature and popular media, often clouded by stormy rhetoric and *ad hominem* arguments that obscure substantive scientific differences (Hall and Guo 2017).

EBM is frequently disregarded based on the perception that it is “obviously” ineffective, since many individuals struggle to lose weight by simply adjusting their caloric balance. EBM proponents respond that this intuition overlooks abundant evidence demonstrating that energy balance reliably influences body mass regardless of macronutrient composition (Hall, 2017). Similarly, studies indicate that overfeeding calories through either increased carbohydrate or fat intake can lead to comparable gains in body fat when other dietary factors are held constant (Horton et al. 1995)^1^.

Meanwhile, CIM is commonly dismissed with the criticism that its central claims violate the laws of thermodynamics. But this critique (perhaps sometimes deliberately) misses the point since it is both theoretically and empirically possible for body fat to increase due to elevated dietary carbohydrate intake even if total calories remain constant (Ludwig and Ebbeling 2018), assuming CIM’s assertions regarding hormone action and alterations in fat cell metabolism triggered by carbohydrates hold true (Hall, 2017). Therefore, the core disagreement between the EBM and CIM centres on the validity of this hormonal and physiological mechanism rather than thermodynamics. Another important area of contention involves the nature of the type of psychological mechanisms controlling eating behaviour: EBM often assumes food intake as largely automatic or unconscious, governed by homeostatic mechanisms, whereas CIM supposes that the relevant food-seeking behaviours may be goal-directed and based on the desire to make up for a perceived shortfall in energy (Magkos et al. 2024).

A useful way to characterize this disagreement is to focus on the direction of causality assumed by competing obesity theories. From this perspective, obesity models can be broadly classified as either “push” or “pull” accounts (Johansen et al. 2025). Push models, anchored by EBM, portray obesity as a process in which an abundant food environment pushes excess energy into storage. Pull models reverse this logic. They propose that changes in metabolism or nutrient partitioning increase physiological demand for energy, pulling calories into storage and stimulating compensatory feeding. The Carbohydrate-Insulin Model represents one influential member of this broader class of fuel-partitioning theories (Friedman et al. 2024; Ludwig et al. 2022). This distinction is useful because it emphasises that the principal disagreement concerns the causal ordering of metabolism and behaviour rather than whether energy conservation ultimately applies.

For our present purposes of further clarifying the logical structure of CIM, it is worth showing in symbols how CIM can explain changes in fat even when energy intake ($\:{E}_{in}$) is kept constant. Suppose total body mass comprises fat * F* and lean body mass * L* that adjusts residually. Assume, for now, that total units of dietary energy come from carbohydrates (* C*) and protein (* P*), so $\:{E}_{in}=C+P=constant$. Suppose that * S* represents the fraction of these calories that are stored as fat. The remaining energy is used for either maintaining and building * L* or expending in activity as energy out ($\:{E}_{out}$) is denoted by *R*.^2^ CIM assumes that the insulin effect, * I*, is an increasing function of carbohydrate intake:

$$\:I=f\left(C\right),\:{f}^{{\prime\:}}>0$$

Where * I* increases fat storage meaning that a higher fraction of calories eaten are stored as fat:

$$\:\varDelta\:F=S\left(I\right)\times\:{E}_{in}-{E}_{out}$$

Assume fat is measured in energy units for simplicity, so that changes in fat mass correspond directly to changes in stored energy. Thus, the fraction of calories consumed converted into fat increases with more carbohydrate intake. Comparing body fat across two times, with $\:{E}_{in}$ and $\:{E}_{out}$ kept constant while altering the quantity of * C* ingested, then we have changes in body fat from initially

$$\:\varDelta\:{F}_{1}={S}_{1}\times\:{E}_{in}-{E}_{out}$$

And then

$$\:\varDelta\:{F}_{2}={S}_{2}\times\:{E}_{in}-{E}_{out}$$

If * C* is increased while * P* is decreased, such that $\:{S}_{2}>{S}_{1}$, then

$$\:{I}_{2}=f\left({C}_{2}\right)>{I}_{1}$$

And the fat storage fraction increases:

$$\:{S}_{2}=S\left({I}_{2}\right)>{S}_{1}$$

To give a change in fat mass as

$$\:\varDelta\:{F}_{2}={S}_{2}\times\:{E}_{in}-{E}_{out}$$

So, even though total body mass remains the same, more calories are stored as fat.

$$\:\varDelta\:{F}_{2}-\varDelta\:{F}_{1}=\left({S}_{2}-{S}_{1}\right)\times\:{E}_{in}>0$$

Thus, even if total calories stay constant, increasing carbohydrate intake raises insulin, which increases the fraction of calories stored as fat, causing fat mass to increase even without eating more calories overall. In other words, the partitioning mechanism considered here allows body fat to increase not because more energy initially enters the body, but because the same incoming energy is distributed differently within it. Such fat accumulation may occur without positive whole-body energy balance when offset by corresponding changes in lean tissue. Sustained increases in total body energy, by contrast, necessarily require cumulative positive energy balance, although the relevant daily increments may be extremely small and difficult to detect directly (Christiansen et al. 2008).

Once changes in nutrient partitioning are allowed to influence hunger and activity, the CIM framework naturally extends into a theory of behaviour. For instance, if fewer calories are available for activity, hunger and fatigue may increase. This could lead to either or both overeating (raising $\:{E}_{in}$) and reducing physical activity ($\:{E}_{out}$). While some proponents of CIM may suppose this change in energy balance either does not occur or is trivial, moderating CIM in combination with EBM allows us to see how altered insulin dynamics caused by high carbohydrate intake could promote obesity through an energy imbalance from overeating total calories.

### Rationally escalating consumption

Once behaviour becomes linked to an underlying biological state, consumption can become self-reinforcing. This feedback naturally lends itself to analysis within a Rational Addiction framework (Becker and Murphy 1988; Jaffe et al. 2019; Stigler and Becker 1977). Here, “addiction” is used in the formal economic sense introduced by Becker and Murphy rather than as a clinical diagnosis. The central idea is that current consumption alters an underlying biological or psychological “stock,” which in turn changes the future marginal benefit of consuming the same commodity. The activity need not be compulsive, unhealthy, or distressing. It need only possess this self-reinforcing structure. Rational addictions can therefore be either harmful or beneficial.

If the idea of “rationally” pursuing a harmful activity seems paradoxical, consider a person who enters a room containing a high concentration of carbon monoxide. Ordinarily, oxygen is transported through the blood by binding to haemoglobin. Carbon monoxide also binds to haemoglobin, reducing the blood’s capacity to transport oxygen. As exposure continues, oxygen deprivation intensifies and the urge to breathe becomes increasingly urgent. Yet each additional breath taken inside the contaminated room increases exposure and further worsens the underlying physiological state.

This example illustrates two important features of Rational Addiction. The first is adjacent complementarity, an economic term meaning that choices made at nearby points in time reinforce one another. In this case, breathing now intensifies the biological demand for another breath shortly afterwards. The second is the distinction between marginal utility and the overall level of utility. Continued exposure leaves the person progressively worse off, yet the immediate marginal value of another breath continues to rise. The locally rewarding action and the globally desirable outcome therefore point in opposite directions. A sufficiently forward-looking person will assign greater weight to escaping the contaminated environment, despite the immediate urgency of breathing. A strongly present-biased person, by contrast, places greater weight on relief from the immediate deprivation. Although this example is deliberately stylised, it shows how each successive action can become more urgently rewarding even as the sequence of actions lowers overall wellbeing.

The same logic applies to dietary consumption. Repeated consumption of energy-dense foods alters specific biological stocks (such as accumulated adipose tissue or baseline insulin secretion as we consider below). Just as carbon monoxide structurally demands more breathing, expanding adipose tissue alters metabolic signalling to increase the future physiological demand for, and appeal of, those same energy-dense foods. The resulting behaviour may be harmful overall while still being understandable as a consistent, forward-looking response to the person’s altered physiological state. The individual does not need to be irrational in the everyday sense; rather, the consequences of earlier consumption have changed the incentives governing later choices. In short, *rational* describes the structure of decision-making, not the desirability of its outcome. Whether an individual resists an immediately rewarding action depends partly on how strongly future costs are valued relative to present benefits.

Obviously, self-reinforcing behaviour need not be harmful. A beneficial Rational Addiction arises when repeated participation increases both the future value of the activity and overall wellbeing. Someone who begins recreational jogging, eating spicy food, or listening to classical music may initially obtain little enjoyment from the activity but, through repeated experience, the activity may become increasingly rewarding (Stigler and Becker 1977). Whether harmful or beneficial, sufficiently strong feedback can generate threshold dynamics: small differences around a critical point may produce either escalation toward a high-consumption state or decline toward a low-consumption state (Jaffe et al. 2019).

### Carbohydrate consumption dynamics

The foregoing discussion provides an intuitive account of self-reinforcing consumption. Formally, Rational Addiction is a model of how consumption of a commodity, * c*, can rationally escalate over time, * t* (Becker and Murphy 1988). Crucially, consumption at different points in time affect the value of more consumption by producing changes in the individual summarized as a stock of “addiction capital” (*A*), such that

$$\:{A}_{t+1}=\left(1-\delta\:\right){A}_{t}+{c}_{t},$$

where $\:\delta\:$ is the rate at which addiction capital naturally depreciates. Utility at any given time is a function such that current consumption is more reinforcing the greater the capital stock the person already has retained from prior consumption:

$${U}_{t}=U({c}_{t},{A}_{t}),\:\frac{\partial\:U}{\partial\:{c}_{t}\partial\:{A}_{t}}>0$$

Crucially, a rational consumer seldom decides based entirely on cravings in the moment; they implicitly weigh the immediate reward of consumption against the future physiological state it will create. This forward-looking calculation is captured by maximizing utility over time:

$$\:max\sum\:_{t=0}^{\infty\:}\beta\:U({c}_{t},{A}_{t})$$

where $\:\beta\:$ represents the individual’s discount factor, capturing the extent to which future utility is valued relative to present utility. Lower values of $\:\beta\:$ imply greater present bias, whereas higher values place more weight on future consequences.

To consider a CIM-based Rational Addiction, let $\:{c}_{t}$ now represent sugar^3^ (carbohydrate) consumption at a given time, and interpret addiction capital at time *t* as modified fat tissue, $\:{F}_{t}$, that increases the marginal utility of sugar consumption (e.g., by increasing cravings or a felt need for more energy intake) and raises the insulin response. Thus, if fat mass is analogous to addiction capital, utility depends on sugar consumption based on how much stored fat the individual has:

$$\:{U}_{t}=U({c}_{t},{F}_{t}),\:\frac{\partial\:U}{\partial\:{c}_{t}\partial\:{F}_{t}}>0$$

Here, $\:S\left(\cdot\:\right)$ includes the efficiency with which consumed energy is stored. If insulin response $\:I\left({F}_{t}\right)$ increases with fat mass, fat dynamics can be represented as:

$$\:{F}_{t+1}=\left(1-{\delta\:}_{F}\right){F}_{t}+S\left(I\left({F}_{t}\right)\right)\cdot\:{c}_{t}$$

Under these assumptions, carbohydrate consumption increases fat mass, while fat mass strengthens the physiological and motivational conditions favouring further carbohydrate consumption. This completes the feedback loop. Short-run gains from consumption may therefore rise alongside its long-run costs. Depending on the strength of the relationships among fat mass, insulin response, and the marginal utility of carbohydrate, the system may contain both stable and unstable equilibria. An exogenous shock, such as a sustained period of unusually high carbohydrate consumption, may increase $\:{F}_{t}$ sufficiently to move the individual beyond an unstable threshold. Below that threshold, fat depreciation and lower consumption return the system toward a leaner, lower-consumption equilibrium. Above it, the reinforcing effects of accumulated fat may draw the system toward a higher stable equilibrium characterised by greater habitual consumption and adiposity.

The central implication is that obesity can become self-reinforcing. Obesity would still require a cumulative positive energy balance, but the Rational Addiction formulation explains how altered nutrient partitioning and biological stocks could generate and maintain the behavioural incentives producing that surplus. Small energy deficits may achieve short-term weight loss without moving the system beyond the threshold separating high- and low-adiposity equilibria. If the underlying biological stock and associated incentives remain largely intact, weight regain may follow. Combining CIM and EBM in this way therefore suggests that even obesity initially driven by altered nutrient partitioning will generally be maintained by correspondingly elevated habitual energy intake.

## The Protein Partitioning Model

The elephant in the room throughout the foregoing discussion is protein. The classic CIM formulation can be re-framed not merely as a story of excess carbohydrate, but as a consequence of dietary protein dilution and the lifestyle factors that determine how effectively dietary protein is translated into functional lean tissue. The same model mentioned earlier for CIM can be modified to consider what happens under different dietary conditions to lean mass. The dynamic is captured as follows:

$$\:{\Delta\:}L=g\left(P,R{E}_{in}\right),\:\:R\equiv\:1-S$$

Where * g* is the energy surplus or deficit gap that is split into either storage as lean body mass or fat, which is a function of * P* (calories from protein), $\:\left(1-S\right)\times\:{E}_{in}$ (calories available for lean mass and energy needs aside from fat storage), and energy expenditure. So, * g(.)* represents net lean mass change after accounting for maintenance costs and turnover (we abstract from explicit timing and conversion efficiencies). Lower protein intake means fewer building blocks to maintain and grow lean mass and calories to support lean tissue, together leading to reduced lean mass or impairment of its maintenance.

Recall that, in a steady state ($\:{\Delta\:}L=0$), lean mass satisfies the equilibrium relation:

$$\:L=h(P,R{E}_{in}),$$

whereas out of equilibrium its dynamics are governed by $\:{\Delta\:}L=g(\cdot\:)$. Under low protein intake, fat could conceivably increase due to higher fat storage while lean mass simultaneously declines. Thus, if changes in fat mass remain captured by

$$\:{\Delta\:}F=S{E}_{in}-{E}_{out},$$

we can reformulate the functional dependencies of the earlier CIM model to show that, holding energy intake and expenditure constant, altering protein intake can nevertheless drive changes in body fat. Let * h* denote the lean-tissue partitioning function, reflecting how protein intake and available energy support lean tissue. Assume that lean mass increases with protein density and energy availability, that systemic insulin sensitivity ($\:{I}_{s}$) increases with functional lean mass, and that the fat-storage fraction (* S*) decreases as insulin sensitivity improves^4^. This gives the functional system

$$\:L=h\left(P,R{E}_{in}\right),\hspace{1em}{I}_{s}=f\left(L\right),\hspace{1em}S=S\left({I}_{s}\right)$$

with

$$\:\frac{\partial\:h}{\partial\:P}>0,\hspace{1em}\frac{\partial\:f}{\partial\:L}>0,\hspace{1em}\frac{\partial\:S}{\partial\:{I}_{s}}<0.$$

.

These assumptions are consistent with a substantial empirical literature showing that increasing protein availability stimulates muscle protein synthesis and promotes the maintenance of lean tissue (Katsanos et al. 2006; Morton et al. 2018; Pasiakos et al. 2013), that greater functional lean mass is associated with improved insulin sensitivity and glucose disposal (Paquin et al., 2024; Srikanthan and Karlamangla 2011; Unni et al., 2009), and that improved insulin sensitivity is generally associated with more favourable nutrient partitioning and reduced metabolically adverse lipid accumulation (Gower and Goss 2015; Turner et al. 2014; Watt and Kraegen 2009). It then follows by the chain rule that:

$$\:\frac{\partial\:S}{\partial\:P}=\frac{\partial\:S}{\partial\:{I}_{s}}\cdot\:\frac{\partial\:{I}_{s}}{\partial\:L}\cdot\:\frac{\partial\:L}{\partial\:P}<0$$

Thus, increasing dietary protein is predicted to lower the fraction of incoming energy partitioned into fat indirectly by increasing lean mass, improving insulin sensitivity, and reducing fat storage. Importantly, this result depends only on the qualitative signs of the relevant partial derivatives rather than the specific functional forms of $\:h(\cdot\:)$, $\:f\left(\cdot\:\right)$, or $\:S\left(\cdot\:\right)$. The model therefore generates qualitative predictions without requiring numerical parameterisation. Lowering fat storage leaves more energy available for maintaining lean tissue. Lower protein intake reverses this process, favouring insulin resistance and further fat storage.

**Fig. 2:**
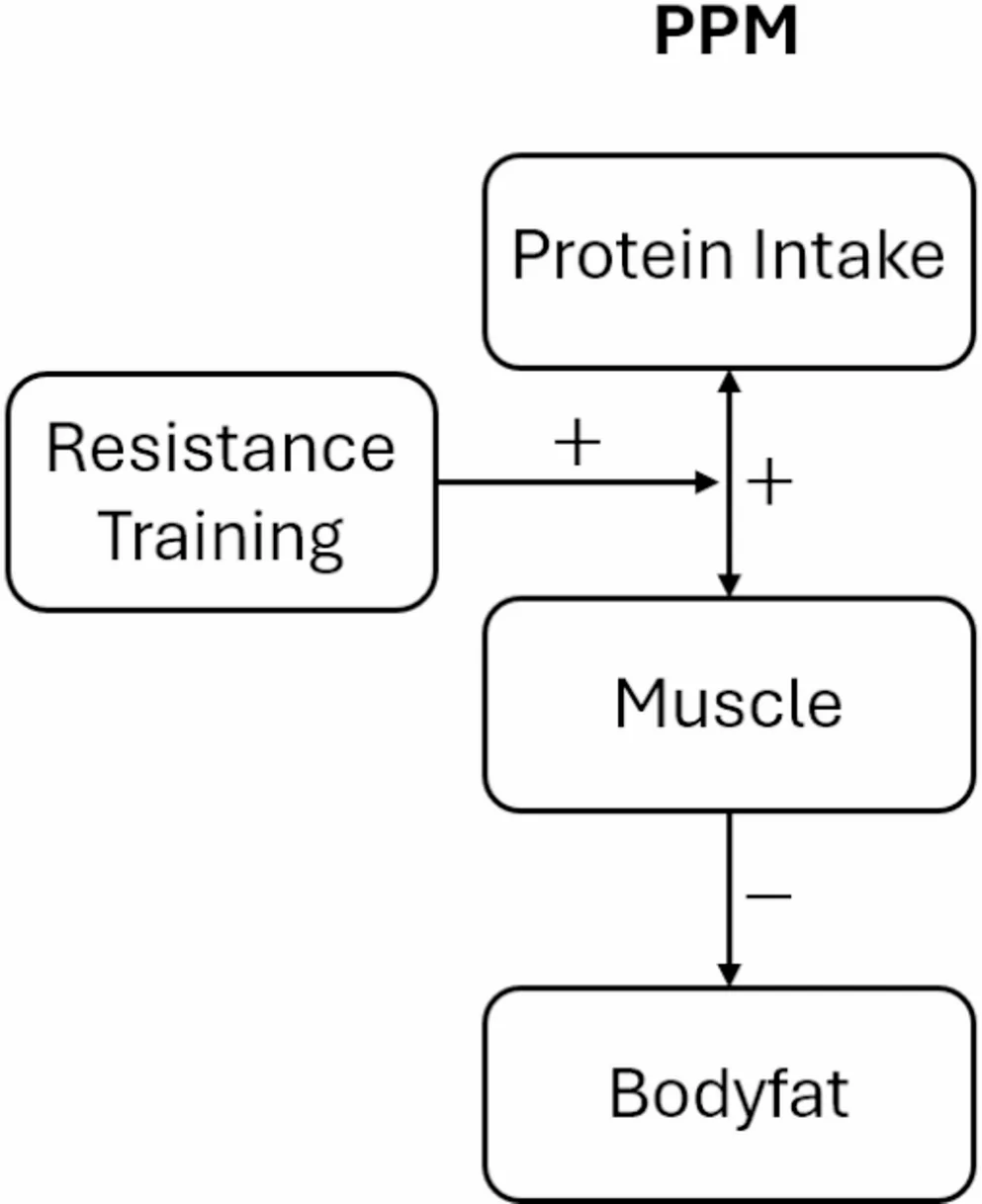
The protein partitioning model. The amount of effective protein available for muscle protein turnover is determined jointly by dietary protein intake and the exercise environment. Positive feedback (+) exists between protein consumption and functional muscle, as greater protein availability supports muscle maintenance and accretion, while greater muscle mass increases physiological demand for protein. Functional muscle exerts an inhibitory (−) influence on fat accumulation by improving nutrient partitioning and reducing the fraction of incoming energy stored as adipose tissue

In other words, whereas CIM attributes fat gain primarily to carbohydrate-driven changes in insulin dynamics, PPM proposes that fat gain may arise from depletion of the biological stock responsible for anchoring energy in lean tissue: functional muscle maintained through sufficient protein intake and mechanical loading. Formally, exercise shifts the functions $\:h\left(\cdot\:\right)$ and $\:S\left(\cdot\:\right)$, rather than entering directly as a separate argument. The same conceptual point is summarized in Fig. 2 and is made with the crucial caveat concerning exercise. Realistically, exercise affects the nature of how nutrients are used in the body (e.g., disposal of excess carbohydrates and thereby smoothing insulin fluctuations), as well as independent effects on insulin function and energy balance. In particular, the degree to which protein is likely translated into lean mass rather than converted into energy through gluconeogenesis depends on what type of physical activity the individual is engaged in. Individuals may expend the same amount of energy through physical activity yet arrive at very different metabolic outcomes. Resistance training acts as a force multiplier on the effects of dietary protein by directing more of that protein toward muscle protein turnover. So, again, we may keep energy expenditure constant, but increasing dietary protein would most likely lead to a change in body composition if the energy expenditure side of the equation involves sufficient resistance training.

If muscle and protein intake are protective against the fat-gain mechanisms proposed by CIM, then it becomes natural to ask how a Rational Addiction to protein might differ from a Rational Addiction to sugar. Both can equally support the Rational Addiction logic, while being addicted to one is incompatible with addiction to the other. Suppose instead that * c* represents consumption of protein-rich foods (such as meat and eggs), with consumption of energy from protein at a given time being $\:{c}_{t}$, while the relevant biological stock is now represented by muscle mass, * M*. Muscle mass increases insulin sensitivity and the marginal utility of protein consumption (e.g., via enhanced muscle maintenance and greater physical capacity). Let utility depend on both protein and muscle mass such that $\:{U}_{t}=U({c}_{t},{M}_{t})$ where $\:\frac{\partial\:U}{\partial\:{c}_{t}\partial\:{M}_{t}}>0$, with the following dynamics:

$$\:{M}_{t+1}=\left(1-{\delta\:}_{M}\right){M}_{t}+h\left({c}_{t},\:R\left({M}_{t}\right)\cdot\:{E}_{in}\right)$$

Where $\:R\left({M}_{t}\right))\cdot\:{E}_{in}$ is the amount of energy not stored as fat, * R* is the fraction of calories available for muscle building and so contributing to accumulation of * M*, and * h* is the partitioning effect that increases with higher protein consumption and more energy that is available and not being used for fat. In other words, the more muscle mass, the greater the physiological demand for protein (higher marginal utility of protein consumption).

Next, consider differences in stock-dependent consumption dynamics between the “Sugar Addiction” (CIM-based) and the “Protein Addiction” (PPM-based) by factoring in two important biological realities. Firstly, muscle is a comparatively “fragile” biological stock. The maintenance of skeletal muscle depends on the continual balance between protein synthesis and breakdown and is highly sensitive to reductions in protein intake and mechanical loading (Mirzoev 2020; Paulussen et al. 2021), whereas adipose tissue is comparatively persistent once accumulated (Spalding et al. 2008). It is therefore reasonable to model the effective depreciation rate of muscle as exceeding that of fat ($\:{\delta\:}_{M}>{\delta\:}_{F}$). Secondly, animals (including humans) regulate protein intake more tightly than either carbohydrate or fat (Simpson and Raubenheimer 2005), with protein being substantially more potent at inducing satiety than carbohydrate (Paddon-Jones et al. 2008; Westerterp-Plantenga et al. 2012). People simply cannot binge on protein as easily as sugar. These constraints suggest that, unlike sugar consumption, increases in protein intake cannot continue indefinitely with increasing muscle mass. Accordingly, we model protein consumption as exhibiting bounded escalation:$\:{c}_{t}\le\:\stackrel{-}{c}+\epsilon{M}_{t}$

**Fig. 3:**
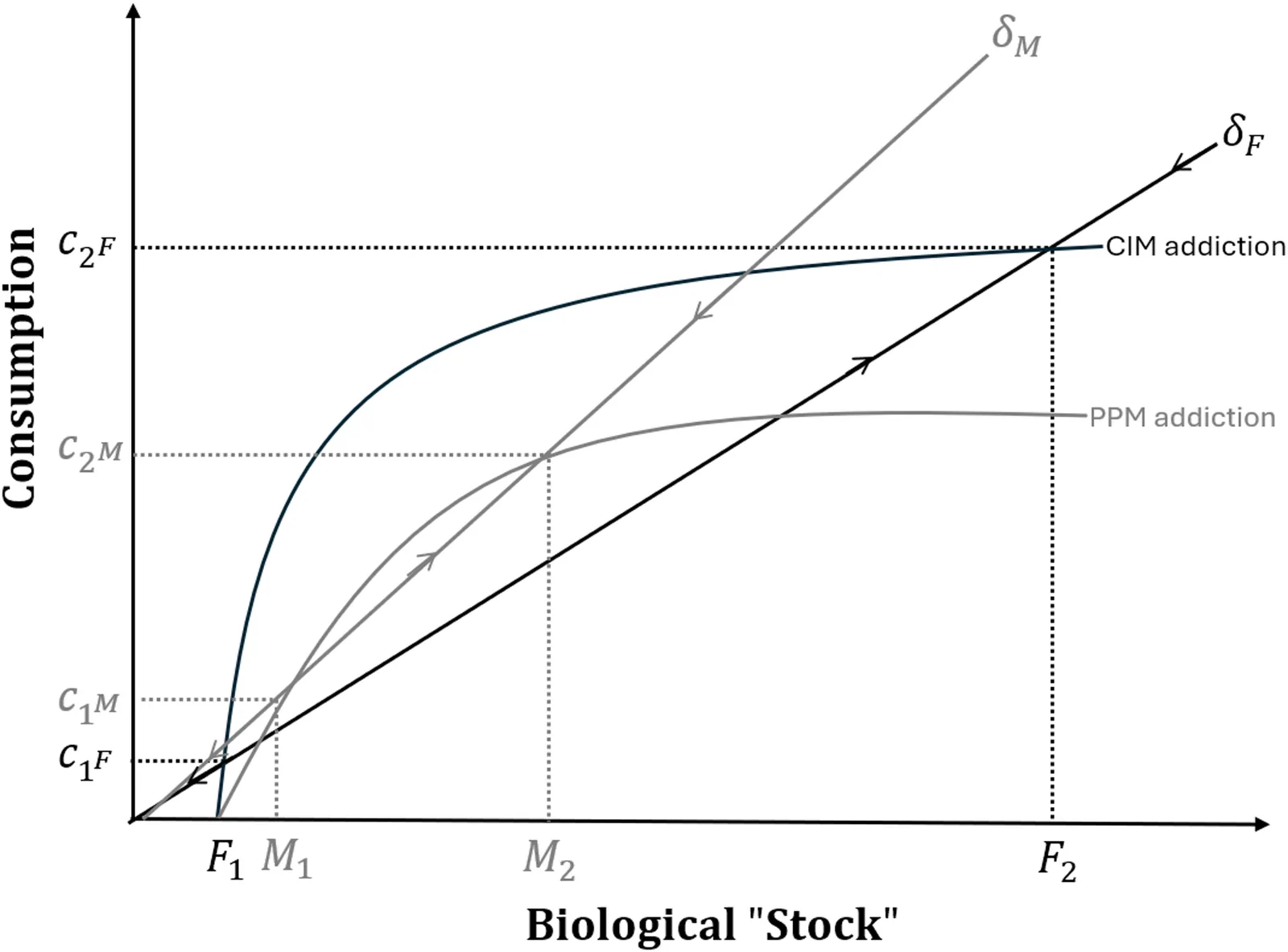
Dual rational consumption paths. Consumption paths for two rational “addictions” are shown (protein in grey and sugar in black). Low steady states for protein consumption dynamic ($\:{M}_{1}{c}_{{1}^{M}}$) and sugar consumption dynamic ($\:{F}_{1}{c}_{{1}^{F}}$) are shown here as unstable, with increases in either consumption of stock driving them into the vicinity of a higher stable steady state. For sugar, this corresponds to a much larger change in stock and regular energy intake, $\:{F}_{2}{c}_{{2}^{F}}$, than for protein, $\:{M}_{2}{c}_{{2}^{M}}$

Where $\:\stackrel{-}{c}$ denotes the physiological upper limit on sustained voluntary protein intake and ε captures the comparatively weak feedback from muscle mass, with $\:\epsilon\:\ll\:1$, reflecting weaker feedback of muscle mass on protein appetite than that observed in the sugar–fat loop. Consequently, the reinforcement loop underlying protein consumption has substantially lower escalation potential and a greater tendency toward bounded, self-limiting dynamics than the corresponding carbohydrate-fat feedback loop. The qualitative differences between the paths of rational consumption to high sugar and to high protein commodities are shown in the Fig. 3. It is thus easy to spiral into obesity through self-reinforcing consumption of sugar, but difficult to reach comparably extreme levels of protein consumption and muscularity. Once fat is accumulated, it does not decline easily without major intervention or efforts to reduce calories. Even sudden cessation of sugar entirely and significant weight loss may not be enough to reach a lower, lasting equilibrium due to the long half-life of fat. Meanwhile, muscle is fragile in that it declines rapidly without sufficient protein intake and resistance exercise. Higher muscle mass raises the marginal utility of protein consumption, but bounded escalation means there are natural constraints on the trajectory of protein-driven consumption and muscle accumulation.

Figure 3 illustrates how these biological asymmetries give rise to fundamentally different long-run consumption dynamics for sugar and protein. These consumption paths are a rising positive function of how much of the biological “stock” an individual has. Steady states are determined where the amount of stock added by a level of consumption is balanced by the relevant rate of depreciation ($\:\delta\:$). Away from these steady states, if the level of consumption means more stock is added than the rate of depreciation, this leads to more stock being gained, while net stock is declining in regions where the curve is under the depreciation line. This is represented by arrows along the depreciation lines showing whether current consumption implies future consumption will rise or fall through its effects on biological stocks. Thus, steady states are stable where flanked by arrows pointing towards them and unstable when flanked by arrows pointing away. For simplicity, assume the same amount of distance in consumption along the *y-*axis of either protein or sugar food corresponds to the same amount of energy intake or grams of protein or carbohydrate, while along the *x*-axis, the same amount of distance represents the same amount of change in muscle (for the protein addiction) and fat (for the sugar addiction). As shown, the higher steady state for the protein corresponds to a lower caloric intake and less extreme change in body composition than the higher steady state for sugar^5^.

It is of course possible to be fat and have much muscle at the same time. However, intrinsic to PPM is that muscle and protein intake tends to interfere with fat deposition and, crucially, to the process by which this fat makes sugar addictive. Therefore, a rational addiction to protein should be protective against a rational addiction to carbohydrate assuming environments have sufficient lean protein options (that is, an individual does not have to eat foods high in both carbohydrate and protein to meet their protein requirements), and that individuals are exercising their muscle tissue in ways that complement the partitioning effect of protein.

## Discussion

Although not intended as an exclusive alternative to CIM or EBM, the present model offers clear and testable predictions that contrast with those of these other frameworks. For instance, CIM posits that a rapid increase in high-glycaemic carbohydrate intake triggers fat gain via insulin spikes – regardless of total energy eaten (Ludwig and Ebbeling 2018). In contrast, PPM predicts that this fat gain need not occur if muscle activity and protein intake are adequate (unless total energy intake is allowed to vary). This divergence arises because protein (and fat) consumption blunts the glycaemic response to sugars while muscle also acts as a key metabolic regulator (Turner et al. 2014; Wolfe 2006) — buffering insulin’s effects and redirecting energy away from fat storage (Srikanthan and Karlamangla 2011).

Within the PPM framework, muscle hypertrophy — stimulated by resistance training and supported by sufficient protein quality and timing — improves insulin sensitivity, increases calorie allocation to muscle, and promotes fat oxidation (Collins et al. 2022; Sartori et al. 2021). Importantly, these benefits can manifest without changes in total body weight, even under high carbohydrate intake (Bosy-Westphal et al. 2015). Conversely, muscle loss due to inadequate protein intake or reduced resistance training diminishes insulin sensitivity and encourages fat accumulation (Beals et al. 2019; Di Meo, Iossa, & Venditty, 2017). The implication is straightforward: PPM therefore emphasizes that changes in body composition are primarily set into motion by perturbations to dietary protein and muscle dynamics, rather than total energy intake (central to EBM) or insulin levels alone (central to CIM).

The central assumptions of PPM concern three linked biological relationships: that protein availability influences functional lean tissue, that functional lean tissue influences systemic insulin sensitivity, and that insulin sensitivity influences nutrient partitioning. The evidence supporting these relationships varies in strength and in the extent to which it maps directly onto the proposed causal structure. The following sections therefore evaluate each component of the model in turn.

### Protein availability and functional lean tissue

The empirical foundation for PPM partly concerns the relationship between protein availability and functional lean tissue ($\:\frac{dL}{dP}$ > 0). Substantial evidence shows that increasing dietary protein intake leads to more favourable body composition changes than merely altering carbohydrate or fat intake. During caloric restriction, higher-protein diets consistently yield greater fat loss and muscle preservation across diverse age groups (Antonio et al. 2020; Aragon et al. 2017; Kanaan et al. 2025). Empirical support for this comes from experimental studies showing that increasing protein or essential amino acid availability increases muscle protein synthesis and improves whole-body protein balance (Katsanos et al. 2006; Pannemans et al. 1997; Pasiakos et al. 2013), together with meta-analytic evidence that higher protein intakes augment gains and preservation of lean mass, especially under resistance-training conditions (Morton et al. 2018; Nunes et al. 2022). This can be contextualised within the classic bioenergetic framework of Gilbert Forbes (1987). Forbes established that baseline energy partitioning between lean and fat mass (the * P*-ratio, $\:\frac{dL}{dW}$) is bounded by initial body fatness, with leaner states favouring lean tissue changes and higher adiposity favouring fat storage. The Protein Partitioning Model does not dismantle Forbes’ thermodynamic baseline but specifies the active macronutrient and mechanical mechanisms that operate upon it. In PPM, dietary protein density, amino acid utilisation efficiency, and resistance exercise provide physiological mechanisms capable of shifting the lean-to-fat accretion ratio beyond that predicted by starting adiposity alone.

Importantly, the role of physical activity may extend beyond its direct energetic consequences. Contemporary work increasingly suggests that exercise does not serve solely as an energy sink through increased expenditure but can also improve appetite regulation and strengthen the coupling between energy intake and expenditure, thereby reducing susceptibility to overconsumption (Blundell and Beaulieu 2023). Thus, the benefits of exercise may arise not only from its direct energetic costs, but also through broader effects on physiological systems governing metabolism, nutrient partitioning, and feeding behaviour. Together, these findings support PPM’s central proposition that protein availability actively influences body-composition dynamics rather than merely reflecting total energy balance.

The second key assumption of PPM is that lean tissue influences the way energy is partitioned within the body, partly through its effects on insulin sensitivity ($\:{I}_{s}$). This dynamic also strengthens PPM’s position *vis-à-vis* CIM. Bosy-Westphal et al. (2015) demonstrate that lean mass modulates resting energy expenditure and metabolic risk during weight cycling. Moreover, revisiting data from the Minnesota starvation experiment, Dulloo and Jacquet (1999) proposed that the body’s ratio of lean mass to fat influences the mobilization of fat and protein during energy deficits. In short, muscle is not merely a passive energy reservoir; it is an active regulator of metabolic partitioning. PPM also fits with evidence that greater muscle mass correlates with lower insulin resistance (Srikanthan and Karlamangla 2011), and that muscle loss (sarcopenia) anticipates metabolic decline and elevated obesity risk (Roh and Choi 2020; Vincent et al. 2012).

The concept of “effective” protein also helps highlight the role of physical activity within PPM. This may be modulated not only through changes in protein quality and timing but also by factors affecting the propensity for protein to be partitioned toward muscle. While there may be many other such factors^6^, for our purposes, exercise is the major example considered here, and physical activity constitutes another key point of divergence among PPM and these other perspectives. CIM regards physical activity as of peripheral importance, viewing carbohydrates—and their insulin-stimulating effects—as central to explaining fat gain (Ludwig and Ebbeling 2018). EBM assigns a more important role to physical activity due to its effect on energy expenditure (“calories out”) regardless of exercise type (Westerterp 2018). PPM offers a different angle, emphasizing physical activity’s role in shaping body composition through interactions with muscle and protein metabolism. Within PPM, the effectiveness of exercise depends less on calories expended than on its capacity to preserve or increase functional lean tissue. For example, aerobic activities such as long-distance running may promote short-term weight loss, but without sufficient protein and resistance training, these often cause disproportionate muscle loss. This muscle loss reduces metabolic rate and increases vulnerability to fat regain, undermining long-term weight management. More generally, PPM suggests that weight loss strategies that do not prioritize muscle preservation—such as those focusing on aerobic exercise, caloric restriction, bariatric surgery, or pharmacological methods such as GLP-1 receptor agonists while neglecting protein — are likely followed by rebound fat gain, with regained weight consisting disproportionately of fat rather than lean mass. Thus, PPM reframes the weight loss goal from simply lowering body weight to improving the quality of lost weight by strategically preserving lean tissue.

Importantly, PPM does not predict that all lean tissue is metabolically equivalent. Muscle quality matters. Mitochondrial dysfunction in muscle, such as due to intramyocellular lipid accumulation (i.e., despite having ostensibly healthy amounts of muscle mass this may be heavily “marbleized” or “fatty” muscle tissue in sedentary individuals), often precedes insulin resistance (Shulman 2000) and so may influence the extent to which lean tissue contributes to insulin sensitivity. Moreover, grip strength — a widely used, albeit crude, indicator of muscle health — is among the strongest predictors of all-cause mortality, cardiovascular events, and functional decline, even after adjusting for age, sex, and BMI (Wu et al. 2017). That maintaining grip strength with age is associated with reduced health risks underscores the metabolic importance of maintaining muscle mass and function (Leong et al. 2015)^7^.

While resistance training alone may not always lead to greater weight loss than aerobic exercise, strong evidence supports PPM’s prediction that combining resistance training with high protein intake produces synergistic benefits in body composition. Studies show this combination preserves more muscle and enhances fat loss during caloric restriction compared to resistance training alone (Mettler, Mitchell, & Tripton, 2010; Sardeli et al. 2018; Weaver et al. 2016; Wycherley et al. 2010). For instance, Weaver et al. (2016) found that obese diabetic patients that combined high-protein diets with a resistance training regime lost more body fat and reduced their waist circumference more than either those on other diets or not exercising in this way. These results support PPM’s central tenet: muscle-preserving interventions require both mechanical (training) and nutritional (protein) inputs, an interaction neglected in calorie- or insulin-centric models.

Taken together, these findings support PPM’s view that protein intake and lean mass are active determinants of metabolic function and body composition rather than passive consequences of energy balance. By contrast, metabolic ward studies have challenged CIM by showing that altering dietary carbohydrate-to-fat ratios can produce equal or greater fat loss under higher-carbohydrate conditions (Hall et al. 2016). These findings are less problematic for PPM because such studies typically hold protein intake constant, thereby preserving the very variable that PPM proposes protects against the adverse metabolic consequences attributed to high carbohydrate intake. Consequently, PPM generates a clear empirical prediction: under conditions of constant energy intake, increasing the effective protein component of the diet should shift body composition toward lean tissue and away from adiposity.

### Protein leverage and protein partitioning

The protein leverage phenomenon follows naturally from the nutrient partitioning dynamics proposed by PPM. When dietary protein density is reduced while total dietary energy remains high, less protein is available to support the maintenance and accretion of functional lean tissue (* L*). Over time, reduced lean tissue may alter metabolic partitioning by reducing insulin sensitivity ($\:{I}_{s}$), thereby increasing the fraction of incoming energy allocated toward adipose storage (* S*) rather than lean tissue maintenance. At the same time, protein-specific physiological demands remain incompletely satisfied, promoting compensatory increases in food intake. Recent human cohort data is consistent with this tissue-mediated dynamic, suggesting that lower dietary protein density strongly drives total energy intake in humans, even while age-related body composition shifts uncouple total energy intake from simple BMI metrics (Honfo et al. 2024).

Thus, PPM proposes that protein leverage emerges from the interaction between appetite regulation and body composition dynamics. Protein dilution may increase energy intake through immediate feedback systems regulating protein adequacy, while also producing longer-term changes in tissue partitioning that favour adiposity. Under this interpretation, overeating on low-protein diets is not simply a consequence of excessive energy availability, but a compensatory response occurring within a system attempting to restore adequate protein allocation while maintaining tissue function. This interpretation is consistent with evidence that dietary protein dilution promotes hyperphagia across diverse species (Raubenheimer and Simpson 2019; Simpson and Raubenheimer 2005; Sørensen et al. 2008), and that increasing dietary protein density can reduce voluntary energy intake. Whether these appetite responses are mediated specifically through the tissue-partitioning mechanisms proposed by PPM remains an important empirical question.

### Nutrient-specific motivation and biological stocks

The Rational Addiction framework has previously been applied to behaviours such as smoking and food consumption but has not been used to model interacting biological stocks such as fat and muscle. CIM (Ludwig and Ebbeling 2018) and the Protein Leverage Hypothesis (Raubenheimer and Simpson 2019; Simpson and Raubenheimer 2005) provide physiological bases supporting components of this model. The “bounded escalation” implies that consumption can increase in response to higher needs (e.g., muscle hypertrophy and repair), but its rate of increase is slow due to physiological constraints related to digestion, absorption, and anabolic capacity (Phillips 2014). Likewise, the concept of a biological “ceiling” on protein intake is well supported by protein’s stronger satiety effects compared to carbohydrate (Leidy et al. 2015; Simpson and Raubenheimer 2005). Sugar consumption can escalate with minimal satiety cues, while muscle accrual from increased protein intake and resistance training is slower and more constrained biologically. Of course, the bounded nature of protein and carbohydrate consumption need not arise solely from the stock dynamics considered here. Acute physiological mechanisms involved in nutrient sensing and appetite regulation, including sensory-specific satiety (Wilkinson and Brunstrom 2016) and nutrient-responsive signals such as FGF21 (Hill et al., 2020; von Holstein-Rathlou et al. 2016), may provide additional negative feedback that constrains escalation and stabilizes consumption trajectories. Crucially, fat accumulation is easier and more rapid through caloric excess, especially from sugar, while taking longer to lose than muscle when the factors that support growth are lessened. This asymmetry lies at the heart of the present approach.

Another important aspect of the model is that it subsumes the principal role of carbohydrates in CIM. This is because muscle mass not only substantially aids in sugar disposal and thereby smooths out insulin spikes (Richter, Sylow, & Hargreaves, 2021), and concurrent consumption of protein with sugar blunts the glycaemic response to the sugar, but muscle tissue and protein metabolism have other independent effects on insulin action and additional metabolic benefits (Wolfe 2006). In other words, protein intake influences energy partitioning through mechanisms that both extend beyond and modify the effects of carbohydrate and adiposity. Together, these mechanisms strengthen the proposed link between muscle maintenance, protein intake, and resistance to fat accumulation. Exogenous shocks, such as a loss in muscle due to prolonged and serious injury or illness, inactivity, aging, or protein under-nutrition, lead to reduced protein appetite through muscle wastage, possibly subsequently contributing to fat gain and more muscle atrophy if the stock of muscle falls sufficiently low to bring the individual within the orbit of a lower equilibrium. This feature fits broadly with views of “catabolic crisis” models of aging and sarcopenia (English and Paddon-Jones 2010).

Although less direct evidence exists for conscious selection of foods by protein versus carbohydrate content or for reward variation by body composition, studies in a wide range of species (including humans) suggest behaviours can differentiate foods differing in macronutrient rewards based on their internal nutritional state (Simpson and Raubenheimer 2012). Evidence is emerging that protein can influence how rewarding foods are perceived. In a study of rats, a low-protein diet increased both preference for protein-rich foods and activation of the nucleus accumbens, a reward- and attention-related brain area, compared to a normal diet (Tomé et al. 2019). The ventral tegmental area (VTA), another key reward-processing region, shows similar effects: protein-deprived rats conditioned to associate flavours with casein or polycose prefer casein and exhibit greater VTA activation in response to it (Chiacchierini et al. 2021). Further studies are required to determine whether other regions involved in learning about and evaluating rewards, such as the ventral pallidum, are similarly modulated by protein status (Ottenheimer et al. 2018). Comparable effects may occur in humans. In obese women, a high-protein meal increased plasma homovanillic acid — a dopamine metabolite linked to VTA activity — and reduced food cravings relative to a lower-protein meal (Hoertel et al. 2014). Similarly, Griffioen-Roose and colleagues (2014) found that women on a low-protein diet showed greater activation of reward-related regions (orbitofrontal cortex, striatum) to food cues and, during ad libitum feeding, selected more protein-rich foods, especially savoury items. Together, these findings suggest that under conditions of protein deprivation, neural reward systems bias behaviour toward protein acquisition.

Re-evaluations of dietary overconsumption suggest that hyperphagia traditionally attributed strictly to “hedonic override” may in fact reflect compensatory homeostatic feeding driven by protein dilution (Spann et al. 2025). That is, animals may overconsume hyperpalatable, energy-dense foods not solely because of intrinsic hedonic reward, but because the marginal reward value of ingested energy remains unfulfilled until protein requirements are met. This distinction underscores a potentially pivotal role for goal-directed incentive learning (Balleine and Dickinson 1998): to appropriately guide instrumental action toward high-protein outcomes during periods of elevated protein demand, the nervous system must accurately encode the specific incentive value of protein relative to changing internal states (Roy et al. 2026a, b). When low-protein, hyperpalatable options dominate the food environment, this mechanism misfires, driving high-volume consumption of non-protein energy that accelerates fat stock accumulation, alters baseline insulin dynamics, and further suppresses the incentive drive needed to maintain functional muscle mass.

In short, far from being restricted to operating as a passive homeostatic reflex, nutrient-specific hungers may dynamically alter reward valuation. Under conditions of muscle turnover or protein depletion, neural reward systems revalue protein-rich outcomes, motivating instrumental choice toward foods that satisfy the body’s underlying structural demands. Within our framework, these findings generate the prediction that protein-rich outcomes should possess a higher dynamic incentive value for individuals with greater functional lean mass or elevated muscle protein turnover (e.g., athletes or individuals undergoing resistance training). Longitudinal dietary intervention studies combining repeated measures of body composition, macronutrient preference, hormonal status, and goal-directed food choice would provide a particularly direct test of this.

### Testable predictions

The theory developed above is ultimately only as useful as its capacity to be empirically falsified. While EBM and CIM have traditionally dominated the experimental agenda in obesity research, PPM opens a distinct frontier. By elevating skeletal muscle from a passive metabolic passenger to an active, causal regulator of both appetite and nutrient partitioning, the model generates a unique set of empirical signatures. Table 1 summarizes six of the clearest opportunities to evaluate these dynamics. Depending on the specific mechanism, these predictions can be tested through controlled animal feeding models, human metabolic ward studies, or longitudinal observational cohorts tracking muscle mass and insulin sensitivity. By “characteristic” of PPM, we do not mean that no conceivable variant of EBM or CIM could accommodate protein or resistance-training effects. Rather, PPM predicts a specific tissue-mediated causal pathway linking protein availability, muscle dynamics, nutrient partitioning, and feeding behaviour.

**Table 1:** Key testable predictions of the protein partitioning model

Prediction	Characterizes PPM	Possible Test
**Isocaloric Partitioning**: Under constant energy intake and expenditure, increasing *effective* protein intake will shift body composition toward lean tissue and away from adipose tissue.	Predicts an active protein-mediated partitioning mechanism. EBM predicts little difference under matched energy balance, while CIM primarily attributes partitioning changes to carbohydrate restriction.	Isocaloric controlled-feeding study varying effective protein intake while monitoring body composition.
**Mechanical Loading Synergy**: At matched total energy expenditure, the effect of higher effective protein intake on body composition will be greater when protein is paired with resistance training than when it is paired with non-resistance exercise.	Predicts that exercise modality influences partitioning through muscle maintenance rather than merely through calories expended.	Energy-expenditure-matched trial crossing exercise modality with protein intake.
**Carbohydrate Buffering**: High carbohydrate intake will not necessarily produce disproportionate fat gain when effective protein intake and resistance training remain sufficient.	Contrasts with strong forms of CIM by predicting that muscle and protein can buffer adverse partitioning effects attributed to carbohydrate intake.	Isocaloric intervention comparing high-carbohydrate diets under conditions of high versus low effective protein and resistance training.
**Muscle as a Causal Variable**: Declines in functional lean tissue will precede and predict subsequent deterioration in insulin sensitivity and increased adiposity.	Treats skeletal muscle as an upstream regulator of energy partitioning rather than a passive consequence of weight change.	Longitudinal study following detraining, bed rest, sarcopenia, ageing, or protein dilution.
**Partitioning-Mediated Protein Leverage**: Protein dilution will increase food intake while simultaneously promoting adiposity through altered nutrient partitioning.	Links protein-driven hyperphagia to changes in lean tissue, insulin sensitivity, and partitioning.	Controlled protein-dilution studies measuring appetite, lean tissue, insulin sensitivity, and adiposity simultaneously.
**Bounded Protein Escalation**: Protein-seeking behaviour can increase with protein need and muscle turnover, but the resulting positive feedback loop will naturally plateau.	Predicts a self-limiting motivational process, contrasting with the greater escalation potential of the carbohydrate-adipose feedback loop.	Longitudinal measurement of protein preference, reward responses, and body composition across varying protein availability and training status.

Collectively, these predictions distinguish PPM from both EBM and CIM by proposing that the trajectory of body composition is fundamentally anchored by functional lean tissue. While certain isolated predictions may overlap with existing theories (such as the hyperphagic response to protein dilution) their synthesis within a single tissue-partitioning framework generates a highly distinctive, testable profile. If they are borne out by future research, they suggest that interventions treating obesity solely as a problem either of passive caloric excess or carbohydrate-driven insulin secretion may not always be targeting the most influential leverage points available.

### Practical implications

If along the right lines, PPM carries several implications for obesity prevention and treatment. Efforts should simultaneously limit sugar exposure to disrupt detrimental metabolic feedback loops and promote environments that support muscle preservation and growth through adequate protein intake and resistance training. While acknowledging sugar addiction and compulsive eating contribute to excessive weight gain and resistance to interventions, PPM suggests that the absence of protein “addiction” may be a critical risk factor predisposing to these patterns. Put differently, EBM and CIM may see obesity prevention as calling for a “defensive” strategy, with recommendations being negative in the sense of stipulating what *not* to do (e.g., do not eat too much or do not eat sugar), and what must be lost (reduce body fat). The protein-addiction approach instead tackles obesity by going on the offensive, recommending what should be *gained* (i.e., lean body mass with likely improved performance capabilities, resilience to injuries and disease through accompanying changes in bone density and so forth) and what should be added (resistance training, protein of appropriate quality and timing, and variety of complementary foods). While speculative, we suspect that reframing the challenge in this way may engage people’s motivations differently and be more empowering.

To forestall a likely misunderstanding, it may seem as though PPM would predict trends in obesity should correlate with rises and falls in protein intake. This is not likely to be the case. We must emphasize the protein relevant to the model is that which feeds lean mass in general and muscle protein in particular, with the latter being related to muscle protein synthesis (MPS) (Phillips and van Loon 2016). At the time of writing, factors that determine MPS include protein quality, meal timing, and the exercise environment (e.g., how much work against resistance muscles have been engaged in) (Layman 2009; Phillips and van Loon 2016). Protein quality depends on amino acid profile and bioavailability, with much higher quantities of low-quality protein sources (e.g., rice and beans) required to trigger MPS than higher quality protein sources (e.g., eggs and beef), with many types of foods that people may eat for their protein content ranging along a spectrum in between (legumes, tofu, fish, chicken, pork, etc.) (Layman 2009; Phillips and van Loon 2016). Timing of a protein meal is also important, with MPS being primed after a period of fasting (e.g., more than a few hours since the last time it was triggered). Grazing patterns of feeding or consuming most of protein in the last meal of the day, are less likely to translate as much protein eaten into lean tissue than protein meals eaten spaced further apart and/or with a large quantity of protein consumed at breakfast (Gwin 2018; Leidy et al. 2011). Thus, protein intake levels may remain constant at a population level while the proportion translated into muscle (rather than simply converted to energy) could decline due to differences in dietary and exercise patterns (Phillips and van Loon 2016). Put simply, stable or even high protein intake need not imply stable *effective* protein intake. Moreover, because the Protein-Leverage effect predicts that protein satiety mechanisms bias people to keep eating over a 24-hour period or so to maintain some constant amount of protein in the diet, people may satisfy their protein hunger through a range of ways that maintain constant protein intake with variable implications for muscle health. Available evidence suggests that protein consumption has overall remained steady in human populations where obesity has risen (Tomé et al. 2019). We would predict there have been trends affecting how much this protein consumption has translated into muscle. For example, it seems reasonable to speculate that, historically, human physical activity likely involved much resistance-type labour and more anaerobic or high intensity bouts of activity (e.g., pursuing prey or fleeing from predators) rather than what is typical in the sorts of modern low-impact exercise characteristic of most leisure and incidental physical activity people engage in today. This may mean our bodies evolved in environments more conducive to developing “protein addictions” that helped maintain muscle mass. A decrease in activities like manual labour and an increasing emphasis on low impact (e.g., aerobic) exercise may be an important factor beyond trends in total energy expenditure. Loss of this drive for muscle maintenance may then have been an underlying cause of rising obesity and metabolic syndrome prevalence.

A final point concerns the “discounting parameter” *β*, representing preference for immediate versus future rewards. In CIM, carbohydrate addiction and elevated *β* exacerbate metabolic dysfunction by prioritizing short-term cravings despite long-term harm (Ludwig and Ebbeling 2018). Conversely, within PPM, investing in muscle through resistance training and appropriate protein intake is predicted to be especially attractive to individuals with relatively high *β*, because these behaviours involve accepting short-term costs in exchange for long-term improvements in health, function, and metabolic resilience. This distinction suggests public health messages emphasizing muscle health and education about “effective” protein consumption may better prevent obesity than focusing solely on conveying the long-term harms of obesity and excessive sugar consumption.

### Limitations

The Protein Partitioning Model is intended as a high-level theoretical framework rather than a quantitatively parameterised simulation of metabolism. Its purpose is to identify general relationships among protein intake, lean tissue, energy partitioning, and feeding behaviour, and to derive qualitative, testable predictions from these relationships. As such, the model intentionally resembles theoretical frameworks such as Hamilton’s rule in evolutionary biology or equilibrium analyses in economics, where the principal contribution lies in identifying parameter-independent constraints rather than estimating particular datasets.

Accordingly, the model assumes only the direction of several empirically supported relationships. In particular, it requires that lean tissue increases with protein availability, that insulin sensitivity tends to increase with functional lean tissue, and that improved insulin sensitivity tends to reduce the fraction of incoming energy partitioned into adipose tissue. The principal conclusions therefore follow from these directional assumptions rather than from any specific parameterisation. The framework does not depend on the precise physiological mechanisms through which these relationships arise. Rather, those mechanisms remain important subjects for empirical investigation, and the model generates predictions about the consequences of their operation without requiring their complete mechanistic specification.

Similarly, the present framework deliberately abstracts from many aspects of human physiology that may prove important for quantitative prediction, including explicit nitrogen balance, organ-specific protein turnover, endocrine regulation beyond insulin sensitivity, differences in protein quality, age-related physiological change, and numerous other determinants of body composition. These omissions should not be interpreted as claims that such processes are unimportant, but as simplifications adopted to produce a tractable theoretical framework capable of generating clear, falsifiable predictions. The purpose of the model is not to estimate the precise magnitude of fat gain, fat loss, or muscle growth under specific conditions. Rather, it aims to identify directional and comparative predictions regarding how changes in protein intake, muscle activity, and lean tissue influence body composition.

The model also uses skeletal muscle mass as a tractable proxy for the component of lean tissue most directly relevant to the proposed behavioural and metabolic feedback loops. While protein metabolism occurs throughout the body, and tissues such as the liver make substantial contributions to whole-body protein turnover, skeletal muscle provides a biologically meaningful state variable linking habitual protein intake, physical activity, insulin sensitivity, and body composition. Importantly, this abstraction should not be interpreted as implying that nitrogen balance is biologically irrelevant or that skeletal muscle is the sole destination of dietary amino acids. Protein turnover occurs across multiple tissues, and nitrogen balance remains a useful aggregate measure of whole-body protein status. PPM focuses on skeletal muscle because it represents the primary dynamic interface connecting voluntary behaviour, mechanical loading, and systemic metabolic regulation. Unlike visceral organs with comparatively fixed turnover, skeletal muscle functions as a large, behaviourally adjustable protein reservoir whose scale and metabolic quality are directly modulated by diet and physical activity. Future extensions may explicitly distinguish among different lean tissues or incorporate nitrogen balance alongside energy balance where greater physiological realism is required.

Similarly, the model should be viewed as complementary to existing approaches emphasizing baseline body-composition dynamics (e.g. Forbes’ partitioning models) or nutrient-specific appetite regulation (e.g. protein leverage theory). PPM is not intended to replace classical partitioning frameworks such as Forbes’ model of the P-ratio, but to specify additional physiological factors that may influence the partitioning coefficient over time. Similarly, PPM incorporates rather than competes with nutrient-specific appetite models by proposing a mechanism through which protein requirements, lean tissue dynamics, and feeding behaviour interact.

A further limitation is that the present model treats insulin sensitivity as a unitary parameter, whereas insulin action is tissue specific. Skeletal muscle insulin sensitivity and adipose-tissue insulin sensitivity may not change in parallel and can have different consequences for metabolic regulation. Future extensions of PPM could distinguish these tissue-specific components to better capture the complex interactions between muscle glucose disposal, adipose lipolysis, and whole-body energy partitioning. The Protein Partitioning Model should therefore be viewed as a conceptual framework for organising existing evidence and generating novel empirical predictions rather than as a comprehensive description of every mechanism regulating body composition. Its value ultimately depends not upon the realism of every simplifying assumption individually, but upon the extent to which its predictions continue to be supported as they are subjected to empirical test.

Finally, the rational addiction component of the model is employed in the formal economic sense introduced by Becker and Murphy, in which current consumption alters future preferences through changes in a biological stock. Its application should not be interpreted as implying any clinical equivalence between feeding and substance abuse. Rather, it offers a compact mathematical apparatus to model forward-looking, goal-directed choice as a function of an evolving biological state variable.

## Summary and conclusion

The Protein Partitioning Model integrates emerging muscle-centric perspectives with established theories of energy balance, nutrient partitioning, and appetite regulation. It proposes that protein availability, functional lean tissue, insulin sensitivity, and feeding behaviour form an interconnected system that influences body composition over time. Rather than treating muscle as a passive consequence of energy balance, it focuses on muscle’s role as an active regulator of nutrient partitioning, feeding behaviour, and long-term body composition. By incorporating these relationships, PPM provides a framework for explaining why protein intake, resistance training, and muscle preservation may be central determinants of long-term metabolic health. The model generates testable predictions regarding obesity prevention and treatment, including the importance of strategies that promote adequate protein intake and preserve functional lean tissue.

## Data Availability

No datasets were generated or analysed during the current study.

## Footnotes

1 Importantly, one often neglected variable in these comparisons is the protein content of the diet (usually kept constant across high carbohydrate versus high fat conditions), which, as we will see below, may significantly modify how energy balance translates into changes in fat and lean mass.

2 𝐸_𝑜𝑢𝑡_ is employed here as a composite term encompassing basal expenditure, activity, thermogenesis, and energy losses through excretory routes (Lund, Gerhart-Hines, & Clemmensen,^2020^).

3 We are focusing on “sugar” because the carbohydrates CIM is most concerned about are those that tend to trigger high glycemic responses.

4 Here, insulin sensitivity ($\:{I}_{s}$) is employed as a high-level systemic variable summarising the aggregate effects of functional lean tissue on whole-body nutrient disposal, glucose regulation, and nutrient partitioning. The model is not intended to distinguish among local tissue-specific insulin responses (which differ across skeletal muscle, adipose tissue, and other organs) but rather captures the established empirical association between greater functional lean mass, improved systemic insulin clearance, and more favourable body-composition outcomes.

5 Factoring in biological realities likely exaggerates the differences shown. For example, eating the same amount of energy from protein as sugar does not lead to as much energy available due to the larger thermic effect of protein metabolism. It is likely very difficult to consume more than a few hundred grams of protein a day due to satiety effects and the larger financial cost, while it is practically easy to achieve this for carbohydrate, implying $\:\stackrel{-}{c}$ or where protein consumption asymptotes are probably very low compared to that for sugar.

6 For instance, mutations in genes such as the myostatin (MSTN) gene, which regulate muscle growth, lead to radically increased rates of muscle accretion and represent a strong test of the model. Animals with MSTN mutations display an endogenous bias to convert more protein into muscle growth, even without exercise, resulting in greater “feed efficiency” — requiring fewer total calories to build and maintain increased lean mass while showing reduced fat accumulation compared to counterparts lacking the mutation (Kahn et al.,^2025^; McPherron and Lee 2002; Ren et al. 2020). Indeed, McPherron and Lee (2002) reported that MTSN deficient mice showed partial protection against developing obesity and insulin resistance.

7 Although some studies have been interpreted as suggesting that training experience attenuates protein responsiveness (e.g., Burd et al.,^2012^), such interpretations require caution. Experienced lifters likely did not experience the same relative physiological stimulus as novices during the standardized exercise protocols used in these studies, making direct comparisons of post-exercise anabolic responses difficult to interpret (Roy,^2026^; Roy and Roy 2026).
